# iMIGS: An innovative AI based prediction system for selecting the best patient-specific glaucoma treatment

**DOI:** 10.1016/j.mex.2023.102209

**Published:** 2023-05-18

**Authors:** Uvais Qidwai, Umair Qidwai, Thurka Sivapalan, Gokulan Ratnarajan

**Affiliations:** a Department of Computer Science & Engineering, Qatar University, Doha, Qatar; b Consultant Ophthalmologist, James Paget University Hospital, Great Yarmouth, UK; c Queen Victoria Hospital NHS Foundation Trust, London, United Kingdom

**Keywords:** MIGS, Minimally invasive glaucoma surgery, Adaptive neuro-fuzzy classification, Personalized medicine, iMIGS: ANFIS-based Prediction System

## Abstract

The use of AI-based techniques in healthcare are becoming more and more common and more disease-specific. Glaucoma is a disorder in eye that causes damage to the optic nerve which can lead to permanent blindness. It is caused by the elevated pressure inside the eye due to the obstruction to the flow of the drainage fluid (aqueous humor). Most recent treatment options involve minimally invasive glaucoma surgery (MIGS) in which a stent is placed to improve drainage of aqueous humor from the eye. Each MIGS surgery has a different mechanism of action, and the relative efficacy and chance of success is dependent on multiple patient-specific factors. Hence the ophthalmologists are faced with the critical question; which method would be better for a specific patient, both in terms of glaucoma control but also taking into consideration patient quality of life? In this paper, an Adaptive Neuro-Fuzzy Inference System (ANFIS) has been developed in the form of a Treatment Advice prediction system that will offer the clinician a suggested MIGS treatment from the baseline clinical parameters. ANFIS was used with a real-world MIGS data set which was a retrospective case series of 372 patients who underwent either of the four MIGS procedures from July 2016 till May 2020 at a single center in the UK.

• Inputs used: Clinical measurements of Age, Visual Acuity, Intraocular Pressure (IOP), and Visual Field, etc.
• Output Classes: iStent, iStent and Endoscopic Cyclophotocoagulation (ICE2), PreserFlo MicroShunt (PMS) and XEN-45).
• Results: The proposed ANFIS system was found to be 91% accurate with high Sensitivity (80%) and Specificity (90%).

## Graphical abstract

**Figure:**
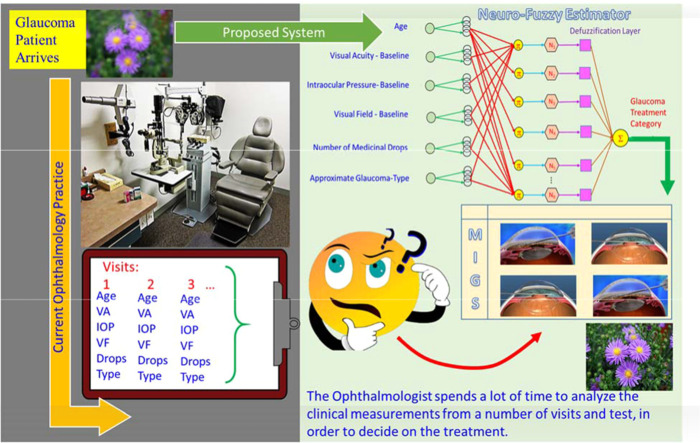

Specifications Table

**Table:** 

Subject area:	Computer Science
More specific subject area:	*Applied AI for Health Informatics.*
Name of your method:	*iMIGS: ANFIS-based Prediction System.*
Name and reference of original method:	https://assets.researchsquare.com/files/rs-664147/v1_covered.pdf?c=1643505460. Initial paper with a similar approach but different type of data and application.
Resource availability:	Audit Data is available on request since it is not available publically.
	Software used: https://www.mathworks.com/help/fuzzy/neuro-adaptive-learning-and-anfis.html.

## Method details

The data used is a retrospective case series of patients who underwent either of the four MIGS procedures (iStent, ICE2, PMS and XEN-45), with or without Phacoemulsification cataract surgery from July 2016 till May 2020 at Queen Victoria Hospital, NHS Foundation Trust, East Grinstead, West Sussex, UK. The project was registered as an audit as it was routine clinical practice, and therefore research and ethics approval was not necessary [1].

All patients attended a baseline visit prior to surgery. Clinical measurements at this visit included intraocular pressure (IOP) using Goldmann Applanation Tonometry, visual field (VF) testing using Humphrey and detailed information was recorded including number of IOP lowering drops in use, best corrected visual acuity (BCVA) in LogMAR units, type of glaucoma (Primary Open Angle Glaucoma (POAG), Secondary open angle glaucoma (SOAG), Primary angle closure glaucoma (PACG), Normal tension glaucoma (NTG), Ocular Hypertension (OHT), etc.), age, gender, lens status, previous glaucoma interventions including Selective Laser Trabeculoplasties (SLT) or Filtration surgeries.

Patients were followed up on 1 week, 1 month, 3 month, 6 month, 12 months, 18 months and 24 months from the date of surgery. On every visit BCVA, IOP and number of drops were documented along with complications or need for further interventions. In visit of 12- and 24-months visual fields were also documented to compare the progression.

For designing a predictive model, the baseline examinations of only the significant variables were used. The main objective of this predictive model is to assist the ophthalmologist when deciding on the most appropriate MIGS treatment. To the best of our knowledge, no such model exists at this point. In addition, the dynamics of the processes are not known analytically such that no simple dynamical model could approximate the given data. Hence, a Machine Learning approach is proposed here where the data patterns are learned through extensive training using the input and output data. The technique used for this work is known as Adaptive Neuro-Fuzzy Inference system (ANFIS) and is explained further in the following section.

## Adaptive neuro-fuzzy inference system (ANFIS)

Artificial Intelligence (AI) is essentially a culmination of a number of algorithms and mathematical systems of equations that, to some extent, mimics the learning and perceiving capabilities that humans have. Most of the conventional AI techniques, such as Naïve Bays, Discriminant Analysis, Parametric Least Square models, Regression models, etc., are limited in their approach to data when the problem under study is extremely non-linear or exhibits complex correlation among the various classes of data under study. Modern data-centric approaches, such as Neuronal Deep Learning, Random Forest, Evolutionary Computing, etc., require a large number of data points in order to capture the patterns in the dataset for appreciable classification. When dealing with clinical datasets, with the exception of images, the actual raw data is usually not too abundant. Simple clinical measurements about specific pathologies and other forms of diseases are not considered to be enough for either the conventional or common data-centric approaches. As can be seen in the previous section, the data being used in this paper has all the limitations that were discussed above, i.e., less than 400 patients with 6 initial clinical readings are the only bases to start with. Most of the neuronal techniques would fail to converge due to scarcity of data. There are 4 output classes to be generated from the pattern classification approach in order to make the system useful for clinical applications. The data has been clinically introduced in the previous section, however, following is another delineation of the same data, only this time it is through the lens of data-science.

Essentially, the system outlines a 6-inputs with 1-output structure where all clinical measurements represent the inputs and possible outcome class (‘Treatment Categories’) represents the output of the system. In its fully trained state, the functionality of the system is designed to act as an advising agent for ophthalmologists in order to assist in better deciding the type of procedure that would be best for the Glaucoma patient in hand. Table 1 shows the values that are the inputs to the system, which represents the usual clinical measurements and initial findings from the physical examination of the patients’ condition:

**Table 1:** Input values for the system with their units and statistical ranges.

	Input Name	Units	Range	Absolute Maximum
1	Age	Years	77.36±3.81	97
2	Visual Acuity	Log mar	0.3 ± 0.25	1.4
3	Intraocular Pressure [IOP]	mmHg	18.54±4.62	40
4	Visual Field	dB	−7.65±7.2	31.11
5	Number of Medicinal drops being taken daily	N/A	2.12±1.08	5
6	Approximate type of Glaucoma	N/A	5.13±1.72	6

Inputs 1 through 5 are actual numerical values and were used accordingly in the learning algorithm. Input 6, and also the Output, which are qualitative values representing Categorical data, were converted into numbers defined as follows:

• Approximate Types of Glaucoma (Input 6)

1. Primary angle-closure glaucoma
2. Normal tension glaucoma
3. Ocular hypertension
4. Primary angle-closure
5. Secondary open-angle glaucoma
6. Primary open-angle glaucoma

• Types of MIGS (Output)

1. iStent and Endoscopic Cyclophotocoagulation
2. PreserFlo MicroShunt
3. XEN
4. iStent inject

No specific reason is associated with the order of the above quantities and were labeled as they appear in the dataset.

**Fig. 1:**
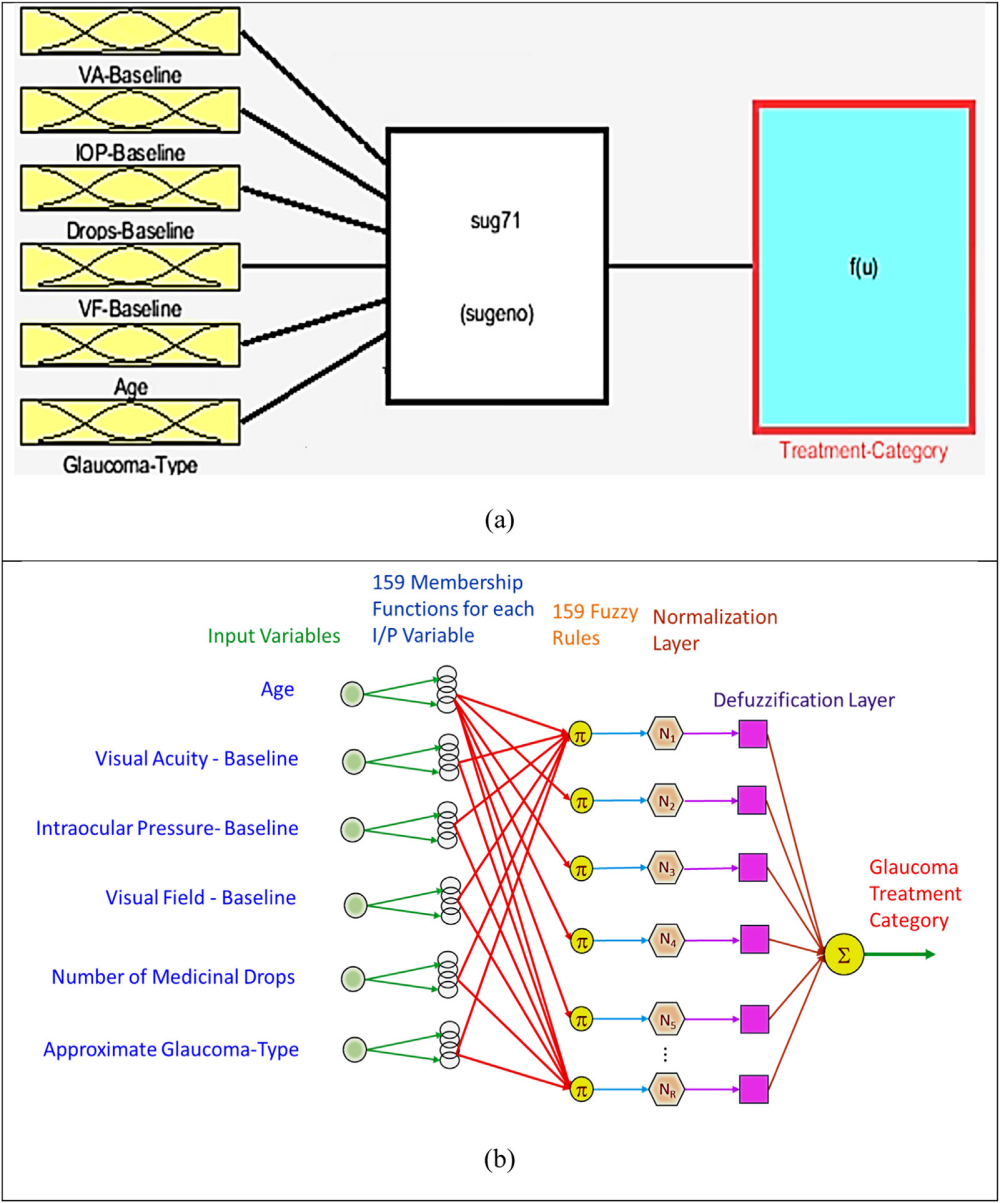
Conceptual layout of the ANFIS algorithm as developed in this work. (a) Block diagram showing input and output channels, (b) Actual pattern of the ANFIS architecture after complete training.

The main AI algorithm used in this work is a hybrid neuronal-clustering and Fuzzy reasoning approach called Adaptive Neuro Fuzzy Inference System (ANFIS) [2], [3], [4], [5], [6], [7]. Based on several years of experience with such algorithms, it has been observed that the technique works very well with small amount of data [[1], [2], [3],^7^]. The shallow neural networks are also claimed to work well with small data sets, but with binary classification. They, usually, fall very short when the multiclass scenario is introduced. ANFIS, on the other hand, circumvents this limitation through a two-step procedure; firstly, the data is clustered based on its similarity scores into a number of clusters. The number of clusters are adjusted by the only user-provided parameter of *Radius of Influence*, which essentially means how many neighborhood samples should be considered within a Euclidean distance of nearness. This can also be understood as the granularity of the data clusters. For the presented work, this was empirically adjusted to 0.15 units. The neuronal layer of ANFIS, creates a large number of clusters; 159 in the presented case. This is a large number of similar groups (almost 2 samples per cluster), but the granularity can help in better classification. Once these clusters of membership are formed, equal number of Fuzzy Rules are made to create 1:1 correspondence with the cluster to its relevant outcome. Fuzzy rules are essentially If-Then-Else type of rules that combines the membership values to the appropriate outcomes in the form of overlapping areas under the membership curves. Such implications of the rules result in a large decision surface composed of possible solutions for a given set of inputs, whose centroid is considered as the final outcome value. Once these centroids are calculated, the output values are *rounded* to nearest integer in order to correspond to the actual numbers selected for representing the outcome classes. This means that a value of the centroid in a range of 1.5 to 2.4 will be thresholder to 2, and so on. Fig. 1 shows the relevant images for depicting the ANFIS structure, and approximate architectural layers in the developed model.

### Rubrics

In order to present the performance of the proposed technique, sample based plots would visually present the closeness of the estimates to the actual values. However, three statistical rubrics of similarity were used to determine the closeness of these estimates to their original data values (i.e., ‘Treatment Categories’). These measures include:

1. *P-value* based significance. {Null hypothesis: Very small or No correlation between the original and estimated sets}. Typically calculated using Pearson's test.
2. Multiclass evaluation measures using *Confusion matrix*. These measures depend upon the usual True Positive (TP), True Negative (TN), False Positive (FP) and False Negative (FN) values which are calculated from the multiclass confusion matrix. These measures include:

a. Sensitivity, hit rate, recall, or true positive rate (TPR)
  TPR=TP/(TP+FN)
b. Specificity or true negative rate (TNR)
  TNR=TN/(TN+FP)
c. Precision or positive predictive value (PPV)
  PPV=TP/(TP+FP)
d. Negative predictive value (NPV)
  NPV=TN/(TN+FN)
e. Fall out or false positive rate (FPR)
  FPR=FP/(FP+TN)
f. False negative rate (FNR)
  FNR=FN/(TP+FN)
g. False discovery rate (FDR)
  FDR=FP/(TP+FP)
h. Overall accuracy for each class (ACC)
  ACC=(TP+TN)/(TP+FP+FN+TN)

**Fig. 2:**
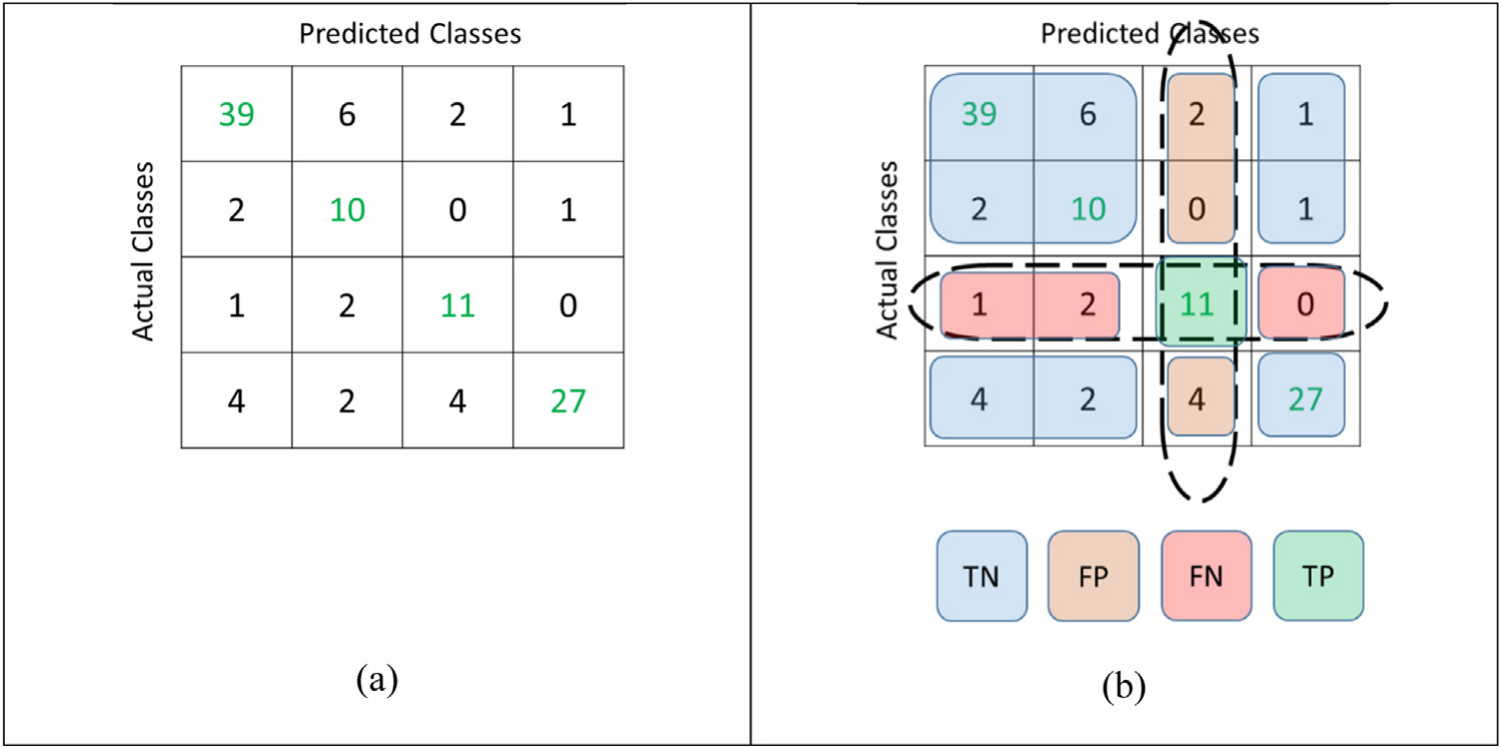
Methodology adopted for finding multiclass TP, FP, TN, and FN values. (a) the Confusion Matrix and (b) selected values for Class 3 such that TP = 11, TN = 92, FP = 6, and FN = 3, where the values are obtained by adding all numbers in the shaded regions of the same color.

Since the presented model is neither a binary classifier, nor an ordinal classifier, therefore the commonly used statistical measures, such as AUC, Sensitivity, Specificity, etc. cannot be calculated for this system in the usual manner without defining specific TP, TN, FP, and FN values. In order to calculate the above-listed measures, the key element is the confusion matrix, which essentially presents the counts of each class being predicted correctly or incorrectly in their respective matrix locations. Each entry in the confusion matrix is obtained when the algorithm is evaluated through the testing data. Each True Class is compared with the predicted class (as predicted by the presented algorithm). Each match is counted as True Positive while each mismatch will correspond to True Negatives, False Positives, and False Negatives. Fig. 2 shows the structure of the confusion matrix and how the TP, TN, FP, and FN are calculated for each class.

## Model development

The overall model was developed using the ANFIS structure and involved the usual machine-learning procedure through using 60% of the available clinical data for training and remaining 40% for testing. Training implies using the randomly selected training samples (where each sample is the set of six input values for each patient) and corresponding output entries in the initialized ANFIS network to train its interconnecting weights (W_L,k_) to ‘learn’ the pattern in the data as a trained model. W_L,k_ represents the value of the *k^th^* weight in L*^th^* layer of the ANFIS structure. The process of model development can be divided into three phases: (1) Preprocessing, (2) Training, and (3) Testing.

### Preprocessing

The original anonymized clinical data is first cleaned for any incomplete sample vector or ambiguous values from clinical point of view. The data is then normalized based on the highest absolute value in that specific range of the input only without considering the normalization of variances. Then 60% of these 6-dimensional samples were randomly selected for training the ANFIS model. Out of these 60%, ANFIS also uses 5% to validate the intermediate training outcomes.

### Training

The randomly selected data is then supplied to the initialized ANFIS structure. The first layer of fully connected neurons train to cluster the input features in Fuzzy memberships using the Neural Learning algorithm. Typically, Gaussian Functions are used to overlap/convolve the data groups and neural weights are trained with the data such that they form the variances and means of the Gaussian membership functions. The resulting set of clusters will group input values according to the nearness within the boundaries of that cluster.

The values obtained through mapping of data with membership functions are then supplied to Fuzzy rules in order to group similar values from the input data into output clusters. This stage involves establishing input cluster to output cluster maps using simple min-max allocated overlaps for each class. A grid partitioning approach is used at this point to establish the 1:1 correspondence between specific input clusters and resulting output maps.

Each time a forward pass is completed, i.e., the weights are applied on the inputs to produce an output, this output is then compared with the desired value of the output and errors values are calculated. These error gradients are then fed back into the network to fine-tune the weights for better fits. The process continues for the complete training space until the rule-base and the neural weights are converged to the final model values.

### Decision surfaces

Once the training is completed, decision surfaces are calculated for a range of all possible input values (with finite increments). Due to the high dimensional nature, i.e., each output is a seven-dimensional model of the input parameters [6-inputs and 1-output], the visualization has to be done with two variables taken at a time thus resulting in 15–3D plots for the output. This is based on the combinations in a set of six variables out of which two are taken at a time. This is given by the Combination formula:

$${}_{r}^{n}C=\;\frac{n!}{r!\;\left(n-r\right)!}=\;\frac{6!}{2!\left(6-2\right)!}=15\;combinations.$$

**Fig. 3:**
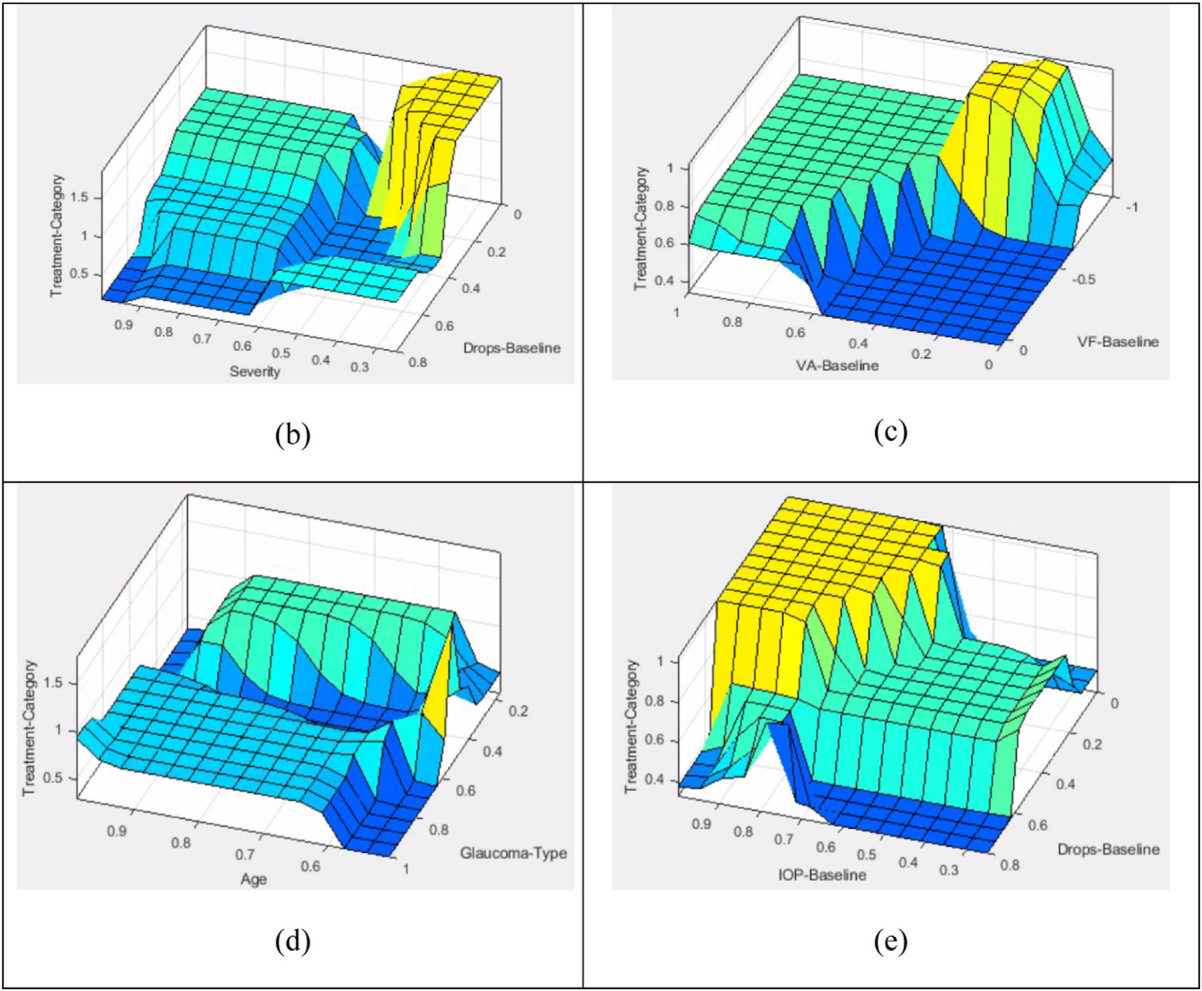
Various decision surfaces that were resulted after the completion of the ANFIS training process. Each surface represents the output as mapped by various two of the seven input variables.

Some of these surfaces are shown in Fig. 3. One can easily appreciate the complexity that has been modelled into these surfaces and that is why the predictability has a lot of confidence in the output values.

## Model validation and analysis

**Fig. 4:**
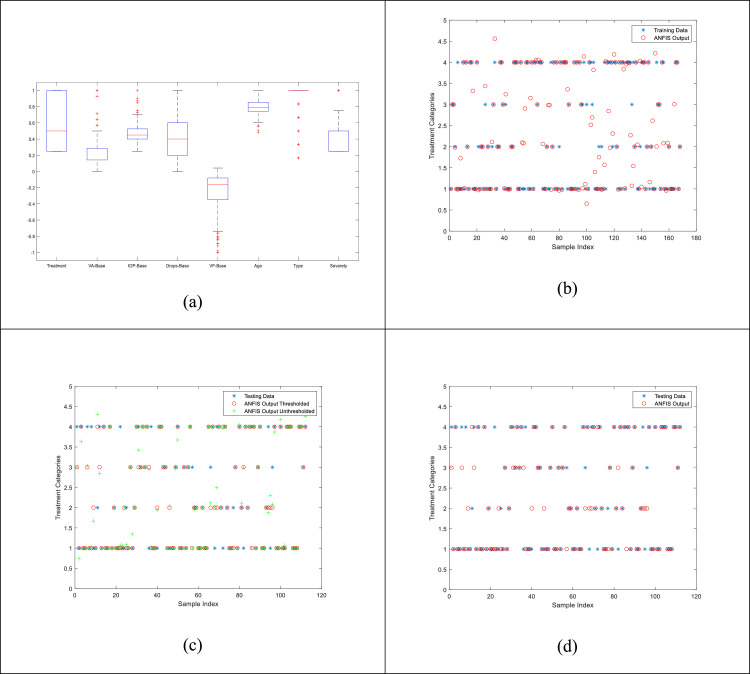
Comparison plots for visual similarity between the original Testing data and the estimated values using the proposed ANFIS system. Blue asterisks represent the original data values, green plus signs represent the un-thresholded raw outputs from ANFIS, and red circles are the thresholded version of the raw outputs from ANFIS. (a) Statistical Box-plot of the data fields used in this work. Each rectangle represents the spread of the specific data field with the lower edge being 25% percentile and the upper edge represents 75%. The red line in each rectangle represents the median of that field, while small horizontal line above and below the boxes represent the standard deviations. The plus sign represents the outlier points for that field, (b) training results, (c) un-thresholded, (d) final thresholded version of the raw estimated outputs of the proposed ANFIS system.

Once the ANFIS model is trained, it is supplied with the remaining 40% of the data that this model has not seen during the training phase. Figs. 4 shows the results of original test data and the corresponding estimated values from the model. Fig. 4(a) depicts the statistical box plots of the data which were used for training and testing of the algorithm. Fig. 4(b)-(d) show the results of training, un-thresholded output classes, and thresholded output classes, respectively. As can be seen that the estimated values are extremely closed to the original values. This shows a converged training of the initial neural architecture and a promising model for the rest of the data. The training data is useful for presenting the fact that the training was kept at a level that over-fitting did not occur. As can be seen that in all practical scenarios, the estimated outputs represent a reasonable degree of agreement with the actual values. Hence, the actual value of the four output classes (ICE2, PMS, XEN, and iStent; represented as 1, 2, 3, and 4 as categorical data) can be estimated based on the baseline data only. This provides a very useful tool for the doctors to make critical decisions in terms of which type of Glaucoma treatment would be best for this patient. Table 2 summarizes the validation statistics between the Actual and the estimated values.

**Table 2:** Table of statistical parameter values used in model validation.

Predicted Classes								
	TPR	TNR	PPV	NPV	FPR	FNR	FDR	ACC
TRUE Classes	0.8125	0.8906	0.8478	0.8636	0.1094	0.1875	0.1522	0.8571
	0.7692	0.8990	0.5000	0.9674	0.1010	0.2308	0.5000	0.8839
	0.7857	0.9388	0.6471	0.9684	0.0612	0.2143	0.3529	0.9196
	0.7297	0.9733	0.9310	0.8795	0.0267	0.2703	0.0690	0.8929

### Comparison with linear-regression, SVM, and shallow NN models

The multiclass classification problem presented in this work proves positively that the nonlinear ANFIS model structure was able to capture the data patterns in order to predict values for the best category of treatment for the given initial measurements of the patient. To investigate further, the feasibility of possibly using standard linear and statistical modeling approaches, neuronal learning approaches, and state-space based modeling approaches, following three techniques were selected based on the simplicity of the design and popularity of usage.

### Multiple input regression-model

This model is based on simple Least Square Estimator (LSE) defined for a multi-input-single-output (MISO) system [8], [9], [10]. This model includes the Age, and the first two samples of VA and MAC are used in the following structure:

$$TreatmentCategory\;\left(TC\right)=a_{1}Age+a_{2}VA+a_{3}VF+a_{4}IOP+a_{5}Drops+a_{6}Type$$

For learning phase, the unknown coefficients are calculated using 60% of the data (randomly selected) and formulated in the following matrix structure:

$$TC_{i}=\left[\begin{array}{cccccc} Age_{i} & VA_{i} & VF_{i} & IOP_{i} & Drops_{i} & Type_{i} \end{array}\right]\left[\begin{array}{c} \begin{array}{c} a_{1}\\ a_{2}\\ a_{3} \end{array}\\ \begin{array}{c} a_{4}\\ a_{5}\\ a_{6} \end{array} \end{array}\right]0\;<\;i\;<\;N$$

**Fig. 5:**
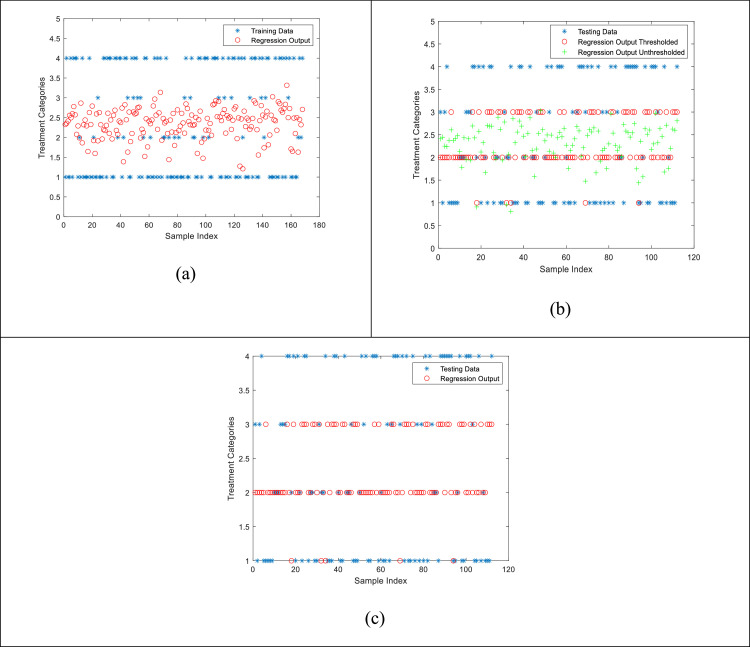
Comparison plots for visual similarity between the original Testing data and the estimated values using the Regression model. Blue asterisks represent the original data values, green plus signs represent the un-thresholded raw outputs from the regression model, and red circles are the thresholded version of the raw outputs from regression model. (a) training results, (b) un-thresholded, (c) final thresholded version of the raw estimated outputs.

Finding the pseudoinverse of the patients’ data and multiplying it from left with the treatment categories column vector gives the unknown coefficients *a*_1_ to *a*_6_. *N* is the total number of observations. These coefficients are then fitted in the original equation with the Testing data (40% of the original data that was kept aside initially) to check for the learning efficiency of the model. These estimated outcomes from the model are then compared with the original testing data outcomes for quantification of the performance. Fig. 5 shows the results from this model.

### Multiclass support vector machine (SVM) model

**Fig. 6:**
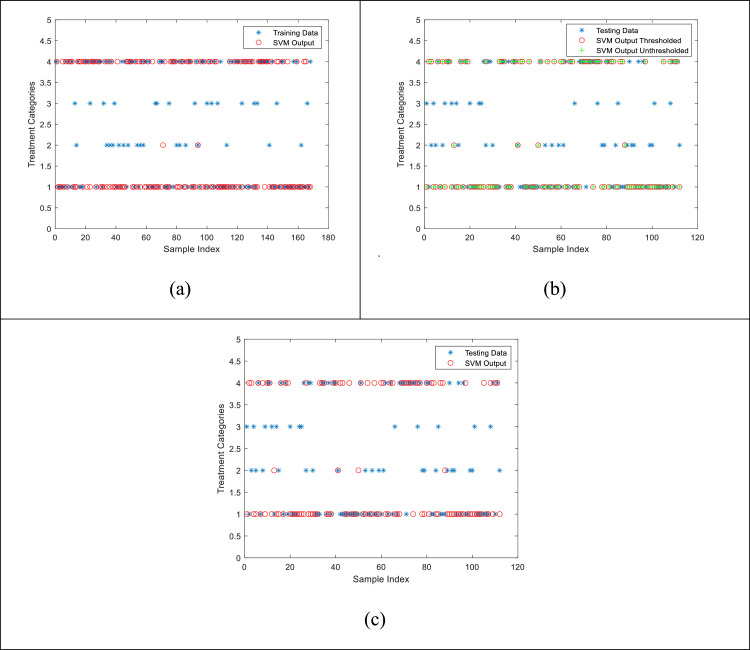
Comparison plots for visual similarity between the original Testing data and the estimated values using the SVM model. Blue asterisks represent the original data values, green plus signs represent the un-thresholded raw outputs from the SVM model, and red circles are the thresholded version of the raw outputs from SVM model. (a) training results, (b) un-thresholded, and (c) final thresholded version of the raw estimated outputs.

This model is based on vector-based multidimensional hyperplane design based on nearness of similar samples in the data space. The orthogonal vectors (called the support vectors) are learned using the training samples and are then tested with the set-aside testing data [11], [12], [13], [14]. SVM has been extensively applied in a very large number of applications and diverse datasets and have been found to work excellently even when the data spread is nonlinear. However, SVM is designed intrinsically for binary classification (i.e., having only two classes in the data), and is not as efficient in case of multiclass scenarios. A twostep strategy is used here for multiclass SVM implementation, called Error Coding and Output Classification (ECOC). First, all the data points are encoded based on their relative distances to the output classes. Then these encoded values are further separated based on their rank scores derived from the statistical distances with the initial classification boundaries. The process is repeated until final convergence is reached. Fig. 6 shows the results from this model.

### Multiclass shallow neural network (NN) model

**Fig. 7:**
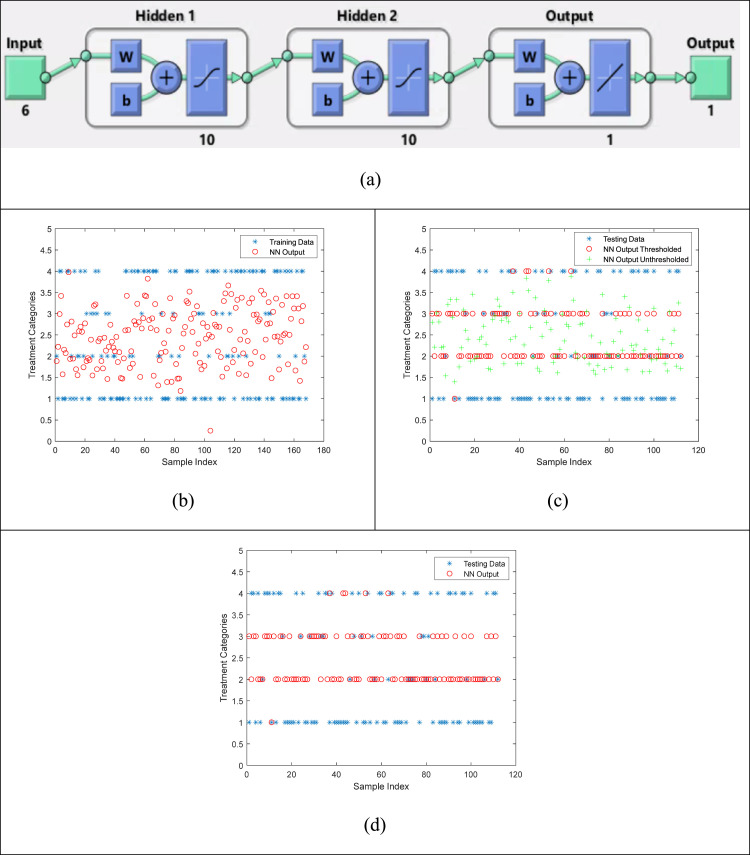
Comparison plots for visual similarity between the original Testing data and the estimated values using the Shallow Neural Network model. Blue asterisks represent the original data values, green plus signs represent the un-thresholded raw outputs from the model, and red circles are the thresholded version of the raw outputs from model. (a) Resultant SNN architecture, (b) training results, (c) un-thresholded, and (d) final thresholded version of the raw estimated outputs.

The concept of orthogonal vectors with respect to the decision boundary is also implemented in multilayer neural networks for solving multiclass classification problems [15], [16], [17], [18], [19]. As part of comparison of the proposed ANFIS model, a Shallow Neural Network (SNN) model is also tested with the same dataset. Fig. 7 shows the results from this model.

**Fig. 8:**
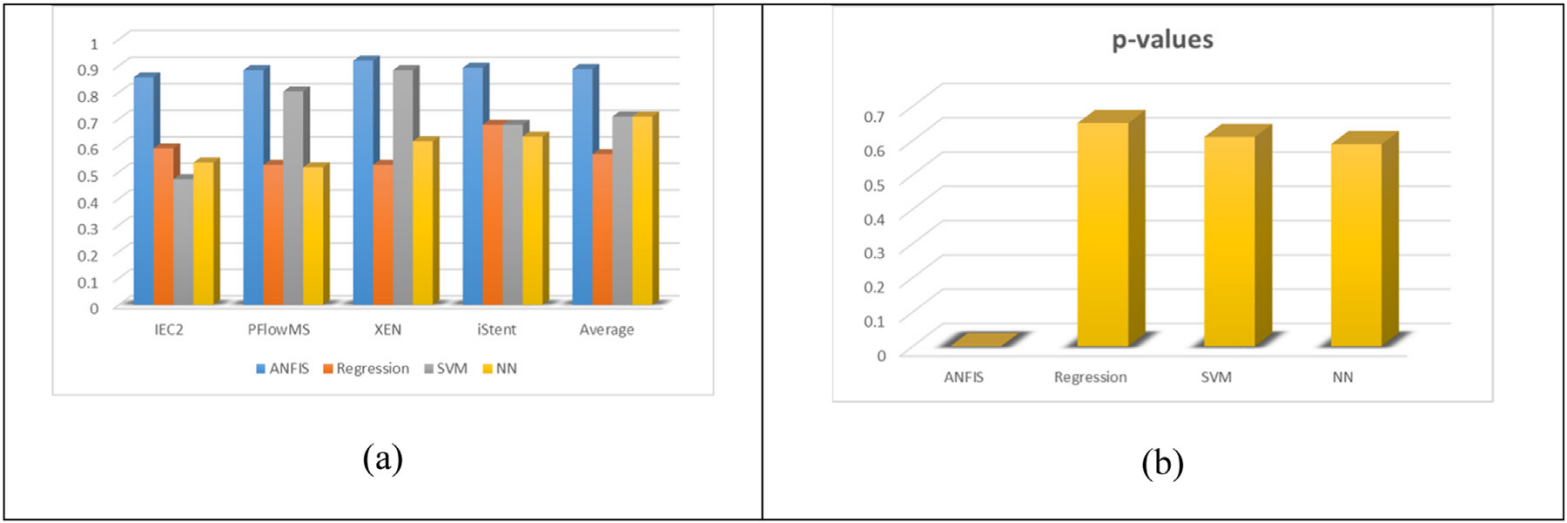
Numerical values for comparisons between the Four techniques; (a) Statistical Accuracy Comparison of the four techniques. An averaged comparison is also shown for the four techniques where the averages are done for all accuracies of the four classes. (b) Comparison of p-values for overall correlation between the actual and the estimated outcomes for all four techniques.

For each of the above comparisons, similar values as those in Table 2 were calculated and are listed in Table 3. As can be seen that in comparison with Table 2, all values in Table 2 show better accuracy, better robustness, recall, specificity, and sensitivity than the three commonly used techniques in similar healthcare problems. Fig. 8 shows the comparison of Regression, SVM, and Shallow NN with ANFIS models in terms of Accuracy. It can be seen that ANFIS outperformed the other methods.

**Table 3:** Comparison of Statistical measures for Regression, SVM, and NN models in order to compare with the ANFIS results.

		Statistical Analysis of Predicted Classes							
		TPR	TNR	PPV	NPV	FPR	FNR	FDR	ACC
Actual Classes 1. IEC2 2. PFlow 3. Xen 4. iStent	**Regression**	0.0233	0.9420	0.2000	0.6075	0.0580	0.9767	0.8000	0.5893
		0.8333	0.4681	0.2308	0.9362	0.5319	0.1667	0.7692	0.5268
		0.1333	0.5876	0.0476	0.8143	0.4124	0.8667	0.9524	0.5268
		0	1	NaN	0.6786	0	1	NaN	0.6786
	**SVM**	0.5625	0.4063	0.4154	0.5532	0.5938	0.4375	0.5846	0.4732
		0.5000	0.9674	0.2500	0.8241	0.0326	0.9500	0.7500	0.8036
		0	1	NaN	0.8839	0	1	NaN	0.8839
		0.6129	0.7037	0.4419	0.8261	0.2963	0.3871	0.5581	0.6786
	**Shallow NN**	0.0189	1	1	0.5315	0	0.9811	0	0.5357
		0.9167	0.4700	0.1719	0.9792	0.5300	0.0833	0.8281	0.5179
		0.4545	0.6337	0.1190	0.9143	0.3663	0.5455	0.8810	0.6161
		0	0.9342	0	0.6636	0.0658	1	1	0.6339

**NaN represents Not a Number which is a way of representing indeterminate quantities.

**Fig. 9:**
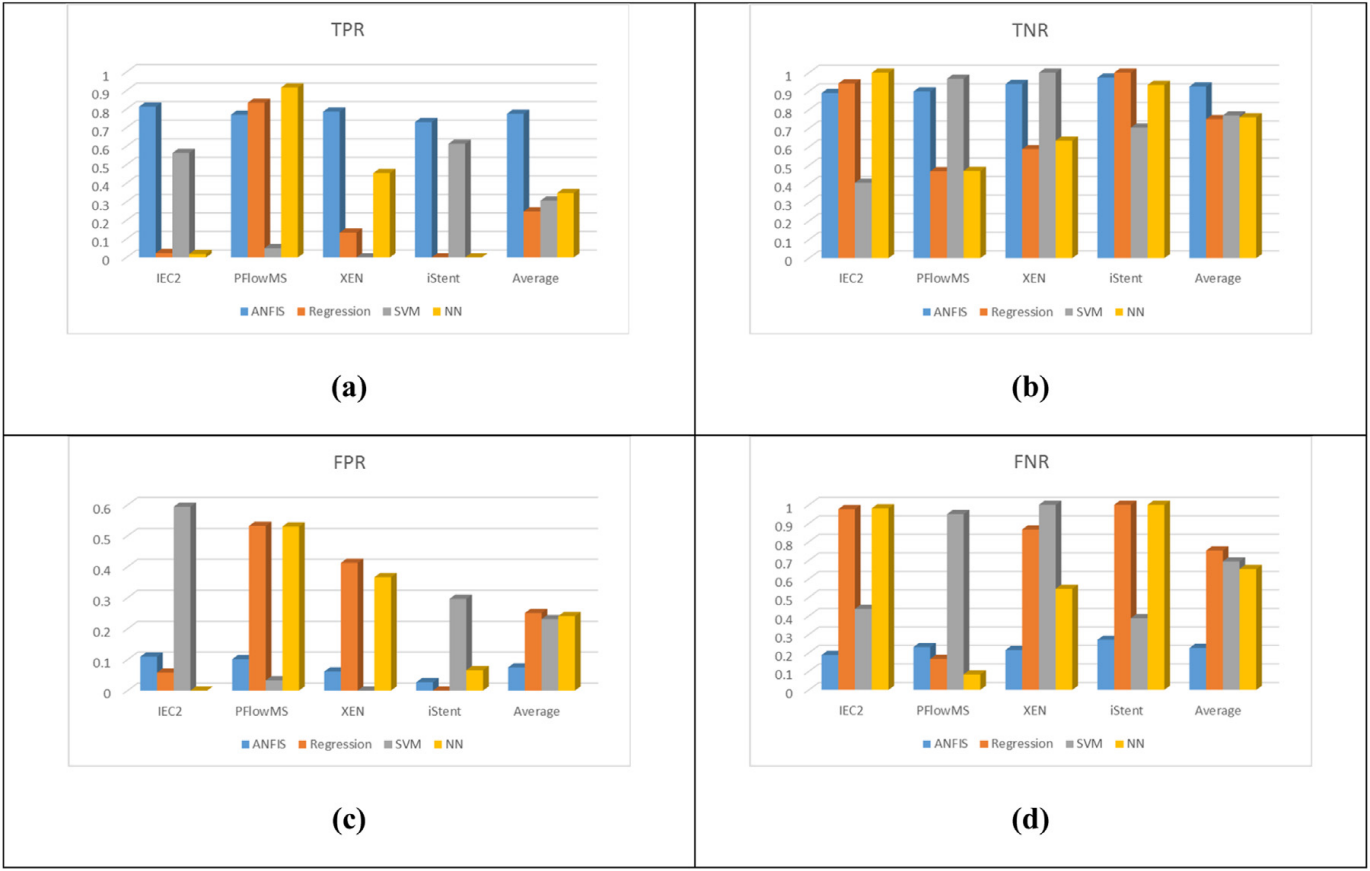
Statistical Comparison of TPR, TNR, FPR, and FNR, for the four techniques (ANFIS, Regression, SVM, and NN models). An averaged comparison is also shown for the four techniques where the averages are calculated in each measurement (TPR, TNR, FPR, FNR) for all values of the four classes. (a) TPR, (b) TNR, (c) FPR, and (d) FNR.

However, for further evaluation, other four measures, TPR, TNR, FPR, and FNR are also plotted for comparison. These are shown in Fig. 9. Ideally, TPR and TNR should be as high as possible, which represent better Sensitivity and Specificity, respectively. On the other hand, FPR and FNR should be as low as possible, which represent the probabilities of false alarm and miss-rate respectively. As can be seen from Fig. 9, on the average, ANFIS model has outperformed all other classifiers and has higher Sensitivity and Specificity, and at the same time low probabilities of false alarm and miss-rate.

## Funding statement

This work is not part of any academic, industrial or governmental funded agency.

## CRediT authorship contribution statement

**Uvais Qidwai:** Conceptualization, Methodology, Software, Validation, Formal analysis, Data curation, Writing – original draft, Writing – review & editing, Visualization. **Umair Qidwai:** Investigation, Resources, Writing – review & editing. **Thurka Sivapalan:** Conceptualization, Investigation, Resources, Writing – original draft, Writing – review & editing, Supervision. **Gokulan Ratnarajan:** Resources, Writing – review & editing, Project administration.

## Declaration of Competing Interest

Mr Ratnarajan is a consultant for Glaukos, Santen and Allergan. All others authors have no conflict of interest.

## Data Availability

Data will be made available on request.
